# Novel Organomineral Complex with Prolonged Antitumor Action

**DOI:** 10.3390/ijms26189205

**Published:** 2025-09-20

**Authors:** Olga Ilinskaya, Galina Yakovleva, Pavel Zelenikhin, Alexey Kolpakov, William Kurdy, Mikhail Glukhov, Igor Sedov, Sergey Kharintsev

**Affiliations:** 1 Microbiology Department, Institute of Fundamental Medicine and Biology of Kazan (Volga-Region) Federal University, Kazan Federal University, Kremlevskaya St., 18, Kazan 420008, Russia; 2 Mineralogy and Lithology Department, Institute of Geology and Oil and Gas Technologies of Kazan (Volga-Region) Federal University, Kazan Federal University, Kremlevskaya St., 4/5, Kazan 420111, Russia; 3 Laboratory of Molecular Basis of Amyloid Formation and Antiamyloid Activity of Chemical Institute Named After A.M. Butlerov of Kazan (Volga-Region) Federal University, Kazan Federal University, Kremlevskaya St., 29, Kazan 420111, Russia; igor_sedov@inbox.ru; 4 Optics and Nanophotonics Department, Institute of Physics of Kazan (Volga-Region) Federal University, Kazan Federal University, Kremlevskaya St., 18, Kazan 420008, Russia

**Keywords:** binase, antitumor activity, zeolite, porosity, prolonged enzyme release, stability, cytotoxicity, human adenocarcinoma cells

## Abstract

Blocking the MAPK pathway is a strategy to stop cancer cells proliferation. Despite all the successes, the acquisition of drug resistance by cells, as well as the mutational status of the downstream protein KRAS, reduces the tumor response to therapy. Ribonuclease binase from *Bacillus pumilus* is among the agents that block this pathway through direct interaction with EGFR and RAS. The present study is aimed at the design, optimization, and characterization of a novel complex based on antitumor binase immobilized on microgranular clinoptilolite-containing rock to ensure its prolonged release in the gastrointestinal tract. A set of modern methods including transmission electron microscopy, scanning electron microscopy, and computed tomography was used to characterize the granularity, porosity and elemental composition of the carrier. The size of binase particles, measured by atomic force microscopy at 7 nm, allows enzyme penetration into meso- and macropores of the carrier. Calorimetric results confirm that binase is stable at high temperatures, even exceeding those in the body, and retains catalytic activity in the model fluids of the gastrointestinal tract. The parameters for processing a natural clinoptilolite-containing rock and the conditions for binase sorption were selected. The gradual release of the enzyme from the carrier lasts over 20 h, which provides cytotoxicity towards human adenocarcinoma cells during movement through the gastrointestinal tract. Thus, for the first time a promising long-acting complex with antitumor and detoxifying properties was successfully created.

## 1. Introduction

Cytotoxic ribonucleases (RNases) represent a novel mechanism-based approach to anticancer therapy. These relatively small proteins selectively attack malignant cells, triggering an apoptotic response and inhibiting protein synthesis. RNases’ therapeutic value was established for onconase (ranpirnase), the RNase derived from the frog *Rana pipiens* (Alfacell, Inc., Somerset, NJ, USA), which has reached phase IIIb confirmatory clinical trial for the treatment of unresectable malignant mesothelioma [1]. Although, at the early 21st century, the tumor cell growth inhibition was confirmed for analogs of onconase from *R. japonica* and *R. catesbiana* oocytes [2], bull semen RNase [3], and human eosinophil cationic protein [4], currently, onconase is the only RNase with an orphan and fast-track drug status granted by the FDA. Prokaryotic RNases also showed anticancer activities [5,6,7,8]. Unlike mammalian RNases, they are not susceptible to omnipresent eukaryotic RNase inhibitor, which protects cytosolic RNA from exogenous RNases [9,10]. It is likely that this property is partially due to oligomeric forms of RNases, including eukaryotic ones, which also avoid inhibition [11]. The most studied representatives of bacillary cytotoxic RNases are barnase from *Bacillus amyloliquefaciens* and binase from *Bacillus intermedius* (the strain was renamed as *B. pumilus*, GenBank Accession No. HQ650161.1). We have isolated and characterized a number of other extracellular RNases of bacilli: balifase of *B. licheniformis* [12] and balnase of *B. altitudinis* [13]. We have found that these secreted RNases exist as natural dimers [14].

The toxic effect of RNases was previously believed to be associated exclusively with the degradation of RNA in actively dividing cancer cells, which predominantly leads to cell death. Currently, a number of cellular components have been identified, the interaction of RNases with which leads to the induction of apoptosis in cancer cells. In over 85% of cancers, the genetic alterations of oncogenes EGFR and RAS, the main components of the mitogen-activated protein kinase (MAPK) signaling cascade, lead to its aberrant activation and subsequent uncontrolled cell proliferation. We have shown that binase inhibits MAPK signaling through direct interaction with EGFR and RAS proteins, which can be considered as good potential therapeutic targets [8,15]. We have also shown that the antiproliferative effect of binase is associated with the blockade of the Ca^2+^-activated K^+^ current in *ras*-transformed, but not in normal, fibroblasts [16]. Thus, not only the catalytic activity but all these effects contribute to the antitumor effect of binase.

Based on these findings, we chose binase to create an antitumor complex with prolonged action. Maintaining the stability of the RNase protein under conditions of its entry into the body, particularly when administered orally, is extremely important, as is the prolonged release of enzyme in gastrointestinal tract.

The biocompatibility of ordered porous silica materials makes them promising candidates for drug delivery systems, particularly in cancer treatment [17]. Clinoptilolite is a naturally occurring zeolite composed of microporous arrangements of silica and alumina tetrahedra, linked through the shared oxygen atoms. It possesses a negatively charged open-frame porous structure, where the negative charge neutralization occurs by cations (Na^+^, K^+^, Mg^+2^, Ca^+2^) capable of ion exchange. The use of derivatives of the active substances and their incorporation into porous materials has shown promising results in protecting the active ingredients from degradation and enabling a controlled release [18]. Naturally occurring zeolites possess anti-inflammatory, antioxidant, and detoxifying properties, which makes them valuable for industrial and medicinal use. Detoxifying effects were established towards radionuclides and heavy metals. The thermally treated natural zeolite from Macicasu (Romania) showed a high capacity to remove Cs^+^ and Sr^2+^ ions from aqueous solutions [19]. Acid treatment of zeolite improves ion exchange capacity and selectivity toward heavy metals greatly enhancing the uptake capacities of Pb^2+^, Cd^2+^, and As^3+^ [20]. Composite materials based on modified clinoptilolite effectively eliminate organic pollutants from contaminated aqueous media [21]. Moreover, the purified clinoptilolite-tuff possesses virucidal activity towards viruses differing in their structure and composition. A wide range of antiviral efficiencies was observed, ranging from up to 99% for Herpes Simplex virus to no activity for Rhinovirus and both bacteriophages [22]. The thermal treatment of zeolite leads to its partial dealumination that is responsible for an increase in the number of clinoptilolite mesopores suitable for incorporating protein molecules [23]. Previously we have shown that among non-treated natural zeolite, clinoptilolite was the least toxic compared to chabazite and natrolite [24]. However, the raw rock cannot be standardized for the pharmaceutical product being created. To do this, at a minimum, it is necessary to process the mineral carrier, evaluate its porosity and granularity, and confirm the absence of cytotoxicity. The present study is aimed at optimizing the designed binase–clinoptilolite complex, namely by the following methods: (i) substantiating the parameters for processing the natural clinoptilolite-containing mineral; (ii) proving the adsorption of binase on the carrier; (iii) visualizing the sizes of binase particles and their correspondence to the pore sizes; (iv) establishing the thermostability of binase structure; (v) maintaining its catalytic activity in gastrointestinal fluids; (vi) supporting the binase cytotoxicity towards human adenocarcinoma cell lines.

## 2. Results

### 2.1. Binase Stability

**Figure 1:**
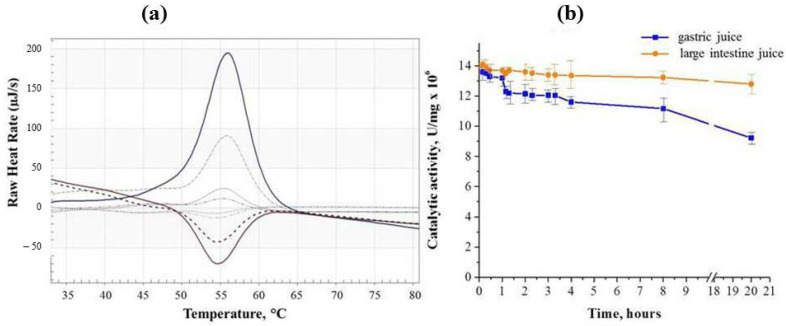
Four repetitive scans of 5 mg/mL solution of binase in deionized water at 2 K/min heating and cooling rates (**a**); dynamics of binase activity preservation in model fluids of the gastrointestinal tract during 20 h of incubation (**b**).

To confirm the stability of binase in the body at elevated temperatures, we investigated the parameters of denaturation and renaturation of the enzyme, as well as the possibility of maintaining its catalytic activity in the gastrointestinal tract. The differential scanning calorimetry (DSC) curves of RNase recorded at 2 K/min scanning rate had a denaturation maximum at 56 °C at both 5 mg/mL and 0.5 mg/mL protein concentration. The enthalpy of denaturation calculated from the peak area was 356 ± 12 kJ/mol. Denaturation of binase is partly reversible. Upon cooling of a solution pre-heated up to 90 °C, renaturation is observed in DSC curves peaking around 54 °C. However, the peak area is more than two times less than the denaturation peak area. Subsequent heating–cooling cycles further reduce the denaturation and renaturation peak areas (Figure 1a).

Calorimetric results confirm that binase is not only stable at high temperatures, even exceeding those in the body, but can also renature with the preservation of structure during multiple heating–cooling cycles. It is especially important that the enzyme maintains its catalytic activity in the gastrointestinal tract for 20 h (Figure 1b). Binase was less stable in gastric juice. In experiments with the main dose of enzyme (100 µg/mL), the loss in activity averaged ~17% after 4 h of incubation, and about 32% of the activity was lost only after 20 h. It takes about 6 to 8 h for compounds to pass through the stomach and small intestine. Then, they enter the large intestine, where they can remain for up to 36 h before leaving the body. Our results indicate that binase gradually released from the carrier will retain a significant part of catalytic activity during transit through the gastrointestinal tract.

### 2.2. Correspondence Between Zeolite Porosity and Binase Size

**Figure 2:**
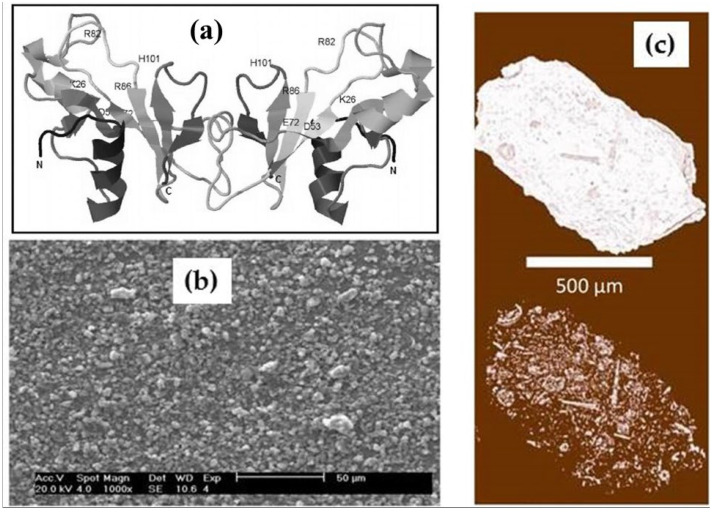
Molecular structure of binase dimer (**a**), granular structure of zeolite carrier (**b**) and porosity of mineral used for binase loading (**c**).

Zeolites are aqueous calcium and sodium aluminosilicates from a subclass of framework silicates, where micropores of 2–15 Å in size make up the primary porosity of the zeolite due to its crystalline structure. The system of secondary porosity consists of meso- and macropores between small zeolite particles. As is known [14], binase is a natural dimer in which the bonds between monomers are stabilized by electrostatic van der Waals forces (Figure 2a). Hydrodynamic radii of binase molecules in solutions are ~2.14 nm [25], which means that the enzyme cannot penetrate into the micropores. The zeolite granules shown in Figure 2b have larger pores and cavities in the micrometer (µm) size range, suitable for protein adsorption (Figure 2c).

**Figure 3:**
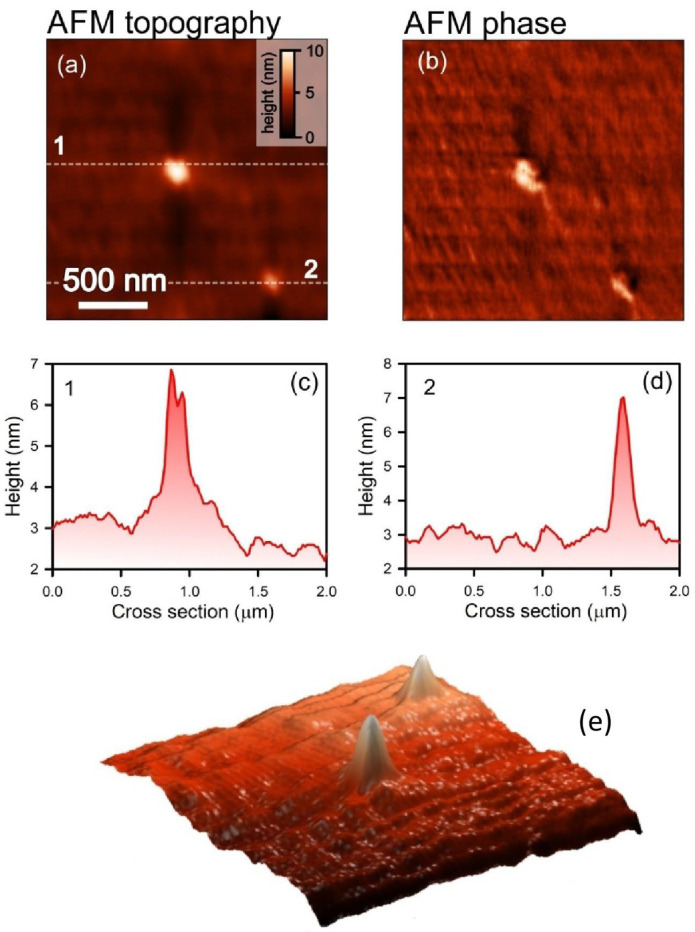
AFM topography of binase protein (**a**) and its phase (**b**) with cross-sections (**c**,**d**) along the dashed straight lines shown in (**a**); 3D AFM visualization of binase (**e**).

However, the probability of binase oligomerization in solution may lead to larger aggregates. We tested this possibility using atomic force microscopy (AFM) (NT-MDT, Zelenograd, Russia). Figure 3 shows micrographs of binase particles in topographic mode, which provides a clear image of the relief of the protein aggregate (Figure 3a) and in phase imaging mode, which reflects variations in the mechanical characteristics of the sample surface (Figure 3b). The cross-sectioning of the samples revealed that binase particles reach a size of 7 nm (Figure 3c,d), which does not prevent their adsorption into meso- and macropores. In the case of binase, we have a homogeneous solution, not a suspension, since the enzyme dissolves well in water. A protein suspension in water is a state in which protein particles, without dissolving, form a heterogeneous mixture with water. Therefore, the distribution of binase particles in the solution can be considered uniform.

### 2.3. Binase Loading and Release

Binase loading into the carrier occurs within 3 h, at which point more than 70% of the enzyme is adsorbed on the zeolite. Increasing the zeolite incubation time with binase solution does not increase adsorption, leading to dynamic equilibrium of the suspension. After drying the loaded samples, we measured the parameters of enzyme release from them into water. If the sample is left in water, then due to the establishment of dynamic equilibrium after 3 h, binase stops leaving the carrier. Therefore, we conducted an experiment with changing the aqueous solution, which was replaced with a new volume of water every hour. The results were promising; in 20 h, we achieved almost complete release of binase from the carrier (Table 1). These data correspond to the process of moving the created organomineral complex through the gastrointestinal tract, where binase will be gradually released from the carrier.

### 2.4. Mechanism Supporting the Penetration of Binase into Zeolite

**Figure 4:**
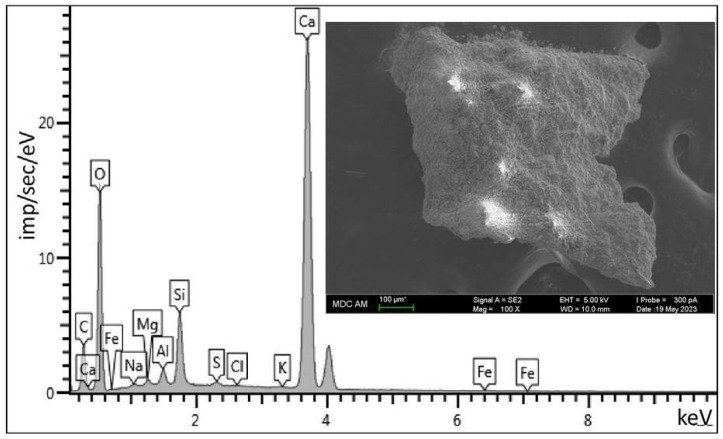
Element composition of zeolite samples used as carriers for binase. The cross in the photo indicates the location of the spectral measurement of the elemental composition of the sample.

Zeolite forms three-dimensional framework structures consisting of SiO_4_ and AlO_4_ tetrahedra. Cations compensate for the excess negative charge of the anionic part of the aluminosilicate skeleton of the zeolite. Because the aluminum centers in the zeolite structure are negatively charged, they require accompanying cations. Zeolites can host cations in the form of metal or non-metal cations. Non-metal cations such as nitrosyl cation NO^+^, ammonium NH^4+^ pyridinium, or protonated (activated) carbon monoxide HCO^+^ have unusual properties/reactivity and are catalytically relevant species for various reactions [26]. We have analyzed the elemental content of the zeolite used in our study (Figure 4) and have shown that calcium is the main cation that balances the negative charge (Table 2).

It has been established that the release of cations into the aqueous medium shifts the pH towards alkaline, from 4.5 for pure distilled water to 7.8 for a zeolite suspension. This process makes the carrier, namely its pores, more anionic, and facilitates the entry of cationic protein into them.

### 2.5. Binase and Zeolite Cytotoxicity Towards Human Adenocarcinoma Cells

**Figure 5:**
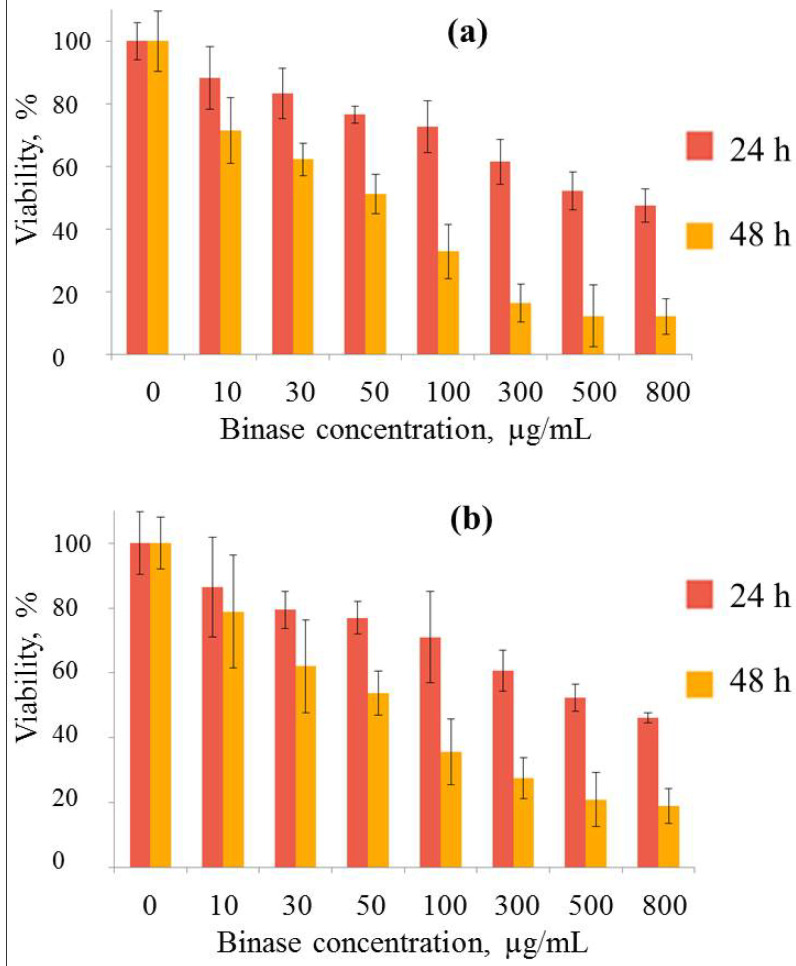
Cytotoxic activity of binase against Hutu 80 (**a**) and Caco-2 (**b**) cells after 24 h and 48 h of incubation.

Binase exhibits strong cytotoxic activity against both lines of adenocarcinoma cells (Figure 5). Cytotoxicity increases slowly with increasing concentration and increases sharply over time. The IC50 value of binase for Hutu 80 cells treated for 48 h was 50.1 ± 0.57 μg/mL, for Caco-2 cells it was a little higher, 60 ± 0.61 μg/mL.

**Figure 6:**
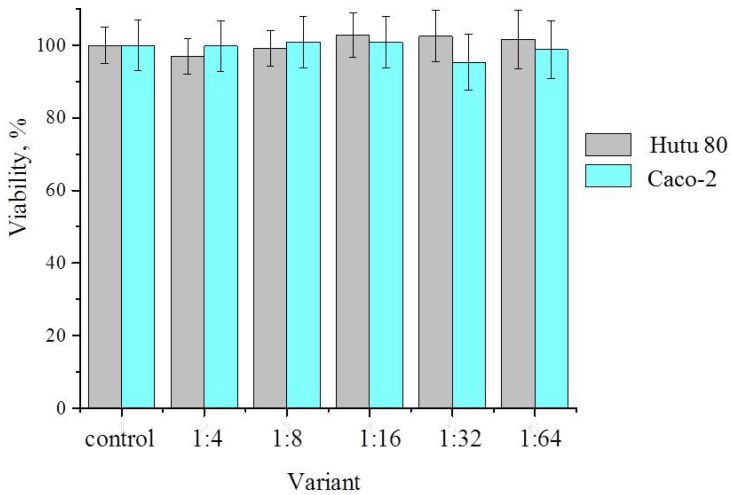
Cytotoxic activity of zeolite extracts against Hutu 80 and Caco-2 cells after 48 h of incubation.

On the other hand, aqueous extracts of zeolite showed no cytotoxicity. The initial extract and its successive dilutions with a nutrient medium for the growth of human intestinal adenocarcinoma cells did not inhibit the growth of tumor cells (Figure 6). These data indicate the safety of the carrier used to create the organomineral complex.

## 3. Discussion

The antitumor activity of RNases has attracted the attention of researchers for several decades. New approaches to cancer therapy are being developed based on RNases. These enzymes are used in combination cancer therapy, a treatment modality that combines two or more therapeutic agents. It was shown that ranpirnase in combination with doxorubicin in clinical trials for the treatment of unresectable malignant mesothelioma had greater anticancer efficacy than doxorubicin alone [27]. The apoptosis-inducing effect of binase in combination with antitumor antibiotic bleomycin on human lung adenocarcinoma cells was higher than individual effect of bleomycin and binase used separately [28]. Barnase conjugated to a therapeutic agent serves as a cytotoxic or secondary module for malignant cell elimination [29].

In our study, we did not consider the detailed mechanism of binase antitumor effects, since they are well known. Binase inhibits MAPK signaling through direct interaction with EGFR and RAS proteins. This effect contributes to the antitumor potential of binase along with its enzymatic activity [8,15]. It is known that binase predominantly attacks tumor cells. Among mouse fibroblasts, v-ras-transformed NIH3T3 cells were sensitive to binase, whereas the growth of non-transformed NIH3T3 cells was not affected [16]. Binase triggers an apoptotic response in cancer cells expressing RAS oncogene, which is mutated in a large percentage of prevalent and deadly malignancies including colorectal cancer. The specific antitumor effect of binase towards RAS-transformed cells is due to its direct binding to RAS protein and inhibition of downstream signaling [8]. Binase decreased viability and induced the selective apoptosis of ovarian cancer cells SKOV3 and OVCAR5. Normal ovarian epithelial cells HOSE1 and HOSE2 were not affected by binase [30]. Previously, we found that binase selectively attacked human A549 alveolar adenocarcinoma cells and triggered an apoptotic response, whereas normal lung epithelial cells LEK were not affected [31].

The multi-target nature of RNases prevents the development of drug resistance, which is considered a major problem of cancer treatment. Today, the drug delivery to tumors is one of the leading research areas in anticancer therapy. For orally administered therapeutics, polysaccharide capsules are used in most cases. However, they quickly dissolve in gastric juice, resulting in immediate release of the active substance. Clinoptilolite is not absorbed and is excreted with the feces. There is no evidence that clinoptilolite will be degraded during its passage through the gastrointestinal tract of animals [32]. When studying the acute toxicity of the zeolite-based prophylactic drug, there were no signs of acute intoxication in mice. When studying chronic toxicity, it was found that inclusion of the drug in the diet of white mice at doses of 1.0 g/kg and 2.0 g/kg of body weight for 2 months did not reveal signs of chronic intoxication, as evidenced by an increase in weight gain of mice in the experimental groups by 1.6–3.4 times compared to the control and 100% survival of mice in all groups [33]. A single injection of zeolite into the stomach of mice in doses from 5 to 20 g per 1 kg of live weight did not cause visible changes in the general condition and behavior of the mice either on the days of zeolite administration or in the following days. Over the course of 21 days of observation, the mice were in good condition and gained weight [34]. Five fractions of native clinoptilolite from the quarry of the Tatarsko-Shatrashanskoye deposit with particle sizes from 0.2 to 5 mm were studied for toxicity towards cow embryo cells of lung epithelium LEK and trachea epithelium TR. It was found that concentrations of up to 300 μg/mL of all zeolite fractions do not have a cytotoxic effect on the cells studied [35]. That is why we chose a natural mineral carrier of the zeolite group to ensure a gradual release of binase from the carrier.

The main rock-forming components of this mineral are clinoptilolite, opal-cristobalite-tridymite phase (OCT phase), clay minerals (montmorillonite), calcite and quartz which account for 90–95% of the tuff volume. OCT phase and montmorillonite, along with clinoptilolite, are natural sorbents [36]. For the first time, we have obtained stable complex of binase immobilized on microgranular clinoptilolite-containing rock. Particularly significant results on the stability of binase were obtained when it was exposed to high temperatures and was incubated in gastric and intestinal juices (Figure 1). A simple method of adsorbing the enzyme from the solution and drying it on a carrier resulted in the production of an organomineral complex from which binase was released gradually (Table 1). We have clearly established the correspondence between the porosity of the carrier and the size of binase, which allows us to conclude that it penetrates into the meso- and macropores of the clinoptilolite-containing rock (Figure 2 and Figure 3). As for the penetration of binase into the carrier, it is facilitated by the release of cations from it and promotes the adsorption of the cationic enzyme (Figure 4, Table 2).

In zootechnics and veterinary medicine, zeolite improves animal health, removes radioactive elements, aflatoxines, and poisons. Zeolite also displays antioxidant, whitening, hemostatic, and anti-diarrheic properties applied in human care [37]. A beneficial effect of clinoptilolite on the concentration profile of metals Al, As, Cd, Co, Pb, Ni, and Sr measured in blood, serum, femur, liver, kidney, small and large intestine, and brain in rats supplemented with the natural clinoptilolite materials was established [38]. The improvements in productive performance and reduction in mycotoxin levels in broilers’ tissues demonstrated a beneficial effect of clinoptilolite zeolite-based mycotoxin binding substance (Minazel^®^ Plus, Patent Co., Misicevo, Serbia) [39]. Activated and micronized zeolites are used as detoxifying agents in humans. Detoxification is attributed to their ability to reduce lipid peroxidation by scavenging free radicals. Mexican smokers administered activated and micronized zeolites had equivalent antioxidant activities as subjects administered vitamin E [40]. Recently, studies on the use of nano zeolite-based disinfectants to clean up contaminated water and soil and using modified and purified nano zeolite to protect health have appeared [41]. A well-characterized medical preparation containing zeolite (Detoxsan^®^ powder) was applied to patients with neuroendocrine tumors suffering from therapy-refractory diarrhea despite receiving standard pharmacotherapy according to the guidelines for carcinoid syndrome and comorbidities. Detoxsan^®^ powder acts as an adsorbent and significantly reduces symptoms of diarrhea [42]. Microporous faujasite zeolite (NaX-FAU) could be used as a drug delivery system to facilitate the oral delivery of poorly water-soluble compounds [43]. The initial results of phase I/IIa trials suggest that oral administration of clinoptilolite may improve lipid profile in individuals with dyslipidemia [44]. The dietary aluminosilicate supplement demonstrated immune-enhancing effects in mice, and clearance effects against porcine circovirus type 2 in experimentally infected pigs as an initial step towards the development of an antibiotic substitute for use in pigs [45]. Synthesized nanosized faujasite demonstrated targeted oxygen delivery and release within brain tumors, representing a biocompatible platform for enhancing tumor oxygenation in anticancer therapy, with significant clinical translation potential [46]. Modern data open new avenues for the use of zeolites as an efficient drug delivery system for drugs with low bioavailability [47].

Now, much attention has been paid to synthetic zeolitic imidazolate frameworks composed of tetrahedrally coordinated transition metal ions (e.g., Fe, Co, Zn) connected by imidazolate linkers which are topologically isomorphic with zeolites. Zeolite-like topologies provide the ability to load various therapeutic and modulating agents into this synthetic carrier [48,49,50]. Nevertheless, the simplicity of natural zeolite obtaining in large quantities, the low cost of its processing methods, high sorption and ion exchange capacity and the absence of toxic effects place it in a leading position in practical application. The various beneficial properties of natural zeolite are being actively studied [51,52]. Zeolite could purify the internal environment of our body, maintain gut microbiota homeostasis for healthy brain activity, and improve the antioxidant and endogenous anti-inflammatory activities, thereby improving the overall wellbeing of the patient. Based on this preclinical research on zeolites was not aimed at finding a new drug, but a food supplement that can improve lifestyle and be combined with traditional pharmacological treatment [53]. Particular attention is paid to zeolites as carriers of various therapeutic agents. Nano zeolite clinoptilolite has the capability of controlled quercetin release until it reaches cancerous cells demonstrating its aptitude for drug delivery [54]. Chemical and physical properties of zeolite allow researchers to develop effective systems for targeted drug delivery applicable in oncology, ophthalmology, dentistry, and orthopedics. Low cytotoxicity, adjustable loading capacity of pores, and effective intracellular targeting confirm the promise of using zeolite and zeolite-like materials in medicine [55]. Therefore, the new organomineral complex with detoxifying and antitumor activity that we have created has a real chance of becoming a promising drug for medical use.

## 4. Materials and Methods

### 4.1. RNase

Binase, the guanyl-preferring RNase from *B. pumilus*, (monomer of 12.2 kDa, 109 amino acid residues, molecular weight 12,213 Da, pI 9.5), was isolated from culture liquid of native binase producer as homogenous protein using the three-step procedure described earlier [13]. The catalytic activity of binase was determined by measurement of high-polymeric yeast RNA hydrolysis products according to modified method of Anfinsen [56].

### 4.2. Zeolite

Clinoptilolite-containing rock was taken from a depth of 18 m in the Tatarsko-Shatrashanskoe deposit, Russia. The mineral was ground to microgranules of less than 30 µm in size using an electric mill (Homaider, Guangzhou, China). High-temperature treatment of the samples was carried out for 30 min at a temperature of 500 °C (EKPS 50 muffle furnace, model 5007, Smolensk, Russia). Thermal activation at this temperature leads to an increase in the porosity of the zeolite due to dehydration and combustion of residual organic matter and is a process widely used in industry and agriculture [57]. Additionally, the zeolite samples were washed twice with 96% ethanol and dried at room temperature. To prepare the zeolite extracts, 1 g sample of sterile zeolites was added to 5 mL of distilled water in 15 mL sterile centrifuge tubes (SPL Life Sciences, Pocheon-si, Korea) and incubated with gentle shaking at 37 °C for 12 h. Then, the samples were centrifuged for 10 min at 4000 rpm (Eppendorf 5702R, Hamburg, Germany), the supernatant was collected and filtered through a sterilizing filter (Corning Inc., Corning, NY, USA) with a pore diameter of 0.22 μm. The extract samples were stored at 4 °C under sterile conditions for no more than three days.

### 4.3. Zeolite Images

Before analyses, microgranular samples were treated with high temperature and washed twice with 96% ethanol. Then, they were sonicated to prevent particle aggregation (10 min, 35 kHz, 130 V, Sapphire, Moscow, Russia). A drop of each sample was placed on a carbon-coated grid and, after ethanol evaporation, was analyzed using transmission electron microscope Hitachi HT7700 Exalens (Hitachi High-Tech Science Corporation, Tokyo, Japan) at a resolution of 1.4 Å. Bright-field images were obtained at an accelerating voltage of 100 kV using an AMT XR-81 camera (AMT Imaging, Woburn, MA, USA).

### 4.4. Elemental Composition of the Carrier

The elemental composition was determined using scanning electron microscopy (Carl Zeiss Merlin with an AZtec X-MAX energy dispersive spectrometer, Oberkochen, Germany). Elemental analysis was carried out at an accelerating voltage of 20 keV and a working interval of 9 mm. A set of reference standards for X-ray microanalysis «Registered Standard No. 8842» of the AZtec program (version 3.3) was used.

### 4.5. Zeolite Porosity Analyses

The pore structure of zeolite samples was analyzed by a computed tomography scanner using a nanofocus tube of a Phoenix V|tome|x S240 (Waygate Technologies, Berchem, Belgium). To create images, the sample was placed in a rotating holder. The images were formed on a digital silicon matrix installed opposite to the X-ray gun as pixel images that are converted into a three-dimensional model, where the brightness characterizes the degree of X-rays absorption as a result of the photoelectric effect and Compton scattering.

### 4.6. Atomic Force Microscopy

The multimode scanning probe microscope NTEGRA PRIMA (NT-MDT, Zelenograd, Russia) was utilized for visualizing the topography and phase of a RNase protein deposited on a glass cover in air. The AFM probes of the “VIT_P” series with resonant frequencies around 350 kHz were used in AFM measurements. The as-prepared sample was measured in tapping mode with a free amplitude of 10–20 nm and a set-point value of A_0_/2.

### 4.7. Enzyme Thermal Denaturation

The studies of binase thermal denaturation were conducted using a differential scanning calorimeter for liquid samples with two 300 μL capillary cells (Nano DSC TA Instruments, New Castle, DE, USA). The sample cell was filled with protein solution in deionized water, and the reference cell was filled with pure deionized water.

### 4.8. Binase Sorption and Desorption

For loading, each sample of treated zeolites (50 mg each) was suspended in 10 mL of aqueous of binase (concentration 1 mg/mL, catalytic activity 14 ± 1 × 10^6^ U/mg), homogenized on a vortex, and subjected to ultrasonication on ice (5 min, 35 kHz, 120 V), after which it was stirred on a shaker for 2 h and precipitated at 4300 g for 5 min. To determine the loading capacity of zeolites, the residual amount of protein in the resulting supernatants was measured by absorption at 280 nm. The values of these indicators for the initial solution of the enzyme were taken as 100%. The precipitates were dried at 50 °C for 30 min, and then 10 mg of samples were suspended in 10 mL of MQ-water and incubated at room temperature, taking 0.5 mL of the supernatant every hour to determine the amount of released protein.

### 4.9. Model Fluids of the Gastrointestinal Tract

A solution simulating the contents of the large intestine had the following composition (g/L): KCl—0.2; NaCl—8; KH_2_PO_4_—0.24; Na_2_HPO_4_—1.44; pH 7.0 [58]. The simulated gastric juice contained (g/L) the following: peptone—8.3; glucose—3.5; NaCl—2.05; KH_2_PO_4_—0.6; CaCl_2_—0.11; KCl—0.37; bile—0.05; lysozyme—0.1 with 1N HCl [59]. Binase was dissolved in these solutions and incubated at 37 °C with periodic measurement of ribonucleolytic activity.

### 4.10. Cell Lines and Cytotoxicity Analysis

Cytotoxicity of binase and mineral carrier was analyzed using the MTT-test with 3-(4,5-dimethylthiazol-2-yl)-2,5-diphenyltetrazolium bromide (Sigma-Aldrich, St. Louis, MO, USA) following the manufacturer’s instructions. Duodenum adenocarcinoma Hutu-80 and colon adenocarcinoma Caco-2 cells were obtained from the Russian cell culture collection of vertebrates (Saint Petersburg, Russia). Cells were cultured at 37 °C in a humidified atmosphere with 5% CO_2_ in DMEM (Gibco, Grand Island, NY, USA) supplemented by 10% fetal calf serum (HyClone, Logan, UT, USA), 100 U/mL penicillin, and 100 U/mL streptomycin. The experiment was carried out in 24-well plates, in which 1.5 × 10^5^ cells per well, containing 500 μL medium, were seeded 24 h before the experiment. After confluence, the medium was replaced with a fresh one containing different binase concentrations or zeolite aqueous extracts in various dilutions. The optical density at a wavelength of 570 nm and a reference wavelength of 630 nm was measured on a BioRadxMark spectrophotometer for plates (USA) after 24 and 48 h. Based on the obtained data, the half-maximal inhibitory concentration of cell viability was calculated using the online AAT Bioquest IC50 Calculator (https://www.aatbio.com/tools/ic50-calculator/) (accessed on 11 December 2024).

### 4.11. Statistics

Statistical analysis and approximation of dependencies were performed using OriginPro 2015 (OriginLab Corp., Northampton, MA, USA). The average of three measurements and the standard deviation were determined. Statistically significant level was taken as *p* ≤ 0.05.

## 5. Conclusions

We have created a promising complex with antitumor and detoxifying properties, providing a prolonged release of the therapeutic agent binase, which blocks the MAPK signaling pathway activated in cancer cells. It has been proven that binase penetrates into the pores of mineral carrier and retains catalytic activity upon release for 20 h, thus ensuring its functional efficiency throughout its movement in the gastrointestinal tract. Binase exhibits high cytotoxic activity against human duodenal adenocarcinoma cells—Hutu 80 and colon adenocarcinoma cells Caco-2. The carrier, high-temperature-treated microgranular clinoptilolite-containing natural rock, is non-cytotoxic. Considering the known sorption activity of clinoptilolite-containing rock, which provides detoxification of heavy metals, radionuclides, and toxins, the created organomineral complex could potentially have a therapeutic effect for patients with oncological diseases. Further research is required to confirm the latter in vivo.

## Figures and Tables

**Table 1:** Binase loading into zeolite and its release with an hourly change in the aqueous solution into which the enzyme is released.

Binase Loading *						
Time, h	0.1	1		2		3
Loading solution, OD_280_	1.40	0.95		0.71		0.40
RNase content in zeolite, %	3.44	34.5		51.0		72.4
Binase release **						
Time, h	0.1	2	3		4	20
Unloading solution, OD_280_	0.16	0.08	0.09		0.15	0.50
Σ(t) OD_280_	0.16	0.24	0.33		0.48	0.98
RNase release, %	10.1	22.9	31.4		45.7	93.3

* For loading, initial content of binase in the solution (1000 µg/mL, D_280_ = 1.45 ± 0.05) was taken for 100%; ** for release, the content of binase in the zeolite was taken for 100%. The standard deviation of three experiments did not exceed 11% (σ ≤ 11%).

**Table 2:** Element composition of zeolite used as a binase carrier.

Element	Weight (%)	Atomic Mass (%)	Standard
O	60.60	78.33	SiO_2_
Na	0.28	0.25	Albite
Mg	0.39	0.33	MgO
Al	0.90	0.69	Al_2_O_3_
Si	3.95	2.91	SiO_2_
S	0.21	0.13	FeS_2_
Cl	0.05	0.03	NaCl
K	0.05	0.02	KBr
Ca	33.47	17.27	Wollastonite
Fe	0.12	0.04	Fe
Sum	100.00	100.00	

## Data Availability

The raw data supporting the conclusions of this article will be made available by the authors on request.

## Abbreviations

The following abbreviations are used in this manuscript:

EGFR	epidermal growth factor receptor
RAS	protein expressed from retrovirus-associated DNA sequences
MAPK	mitogen-activated protein kinase
DSC	differential scanning calorimetry
AFM	atomic force microscopy
